# A coordinate-based ALE functional MRI meta-analysis of brain activation during verbal fluency tasks in healthy control subjects

**DOI:** 10.1186/1471-2202-15-19

**Published:** 2014-01-24

**Authors:** Stefanie Wagner, Alexandra Sebastian, Klaus Lieb, Oliver Tüscher, André Tadić

**Affiliations:** 1 Department of Psychiatry and Psychotherapy, University Medical Centre Mainz, Untere Zahlbacher Str. 8, Mainz, Germany

**Keywords:** fMRI, Coordinate-based activation likelihood estimation (ALE), Meta-analysis, Verbal fluency, Healthy controls

## Abstract

**Background:**

The processing of verbal fluency tasks relies on the coordinated activity of a number of brain areas, particularly in the frontal and temporal lobes of the left hemisphere. Recent studies using functional magnetic resonance imaging (fMRI) to study the neural networks subserving verbal fluency functions have yielded divergent results especially with respect to a parcellation of the inferior frontal gyrus for phonemic and semantic verbal fluency. We conducted a coordinate-based activation likelihood estimation (ALE) meta-analysis on brain activation during the processing of phonemic and semantic verbal fluency tasks involving 28 individual studies with 490 healthy volunteers.

**Results:**

For phonemic as well as for semantic verbal fluency, the most prominent clusters of brain activation were found in the left inferior/middle frontal gyrus (LIFG/MIFG) and the anterior cingulate gyrus. BA 44 was only involved in the processing of phonemic verbal fluency tasks, BA 45 and 47 in the processing of phonemic and semantic fluency tasks.

**Conclusions:**

Our comparison of brain activation during the execution of either phonemic or semantic verbal fluency tasks revealed evidence for spatially different activation in BA 44, but not other regions of the LIFG/LMFG (BA 9, 45, 47) during phonemic and semantic verbal fluency processing.

## Background

Verbal fluency constitutes an executive function which is impaired in various neurological and psychiatric disorders. Tests of verbal fluency are amongst the most widely used measures to assess executive functioning [1]. These tests assess the ability to generate words [2]. The pre-determined categories of verbal fluency tasks can be phonemic or semantic in nature [1]. In standard clinical versions, subjects are given one minute to generate object names from a given category (semantic fluency) or words beginning with a specific letter (phonemic fluency).

Neuroimaging investigations have shown that verbal fluency relies on the coordinated activity of a number of brain areas, particularly in the frontal and temporal lobes of the left hemisphere. Damage to the left frontal lobe, especially to the left inferior frontal gyrus (LIFG) has consistently been shown to impair verbal fluency performance [3-5]. Findings from previous studies suggest that phonemic verbal fluency relies on a partially different network of brain regions [6,7]. Studies have shown that frontal lobe damage results in impairment to phonemic fluency, whereas temporal lobe damages rather impair semantic than phonemic verbal fluency [8-10]. Furthermore, a functional dissociation of the LIFG along semantic-phonological domain lines has been suggested [4,6,11,12]. The anterior-ventral LIFG (BA 45, 47) is supposed to be specifically involved in the processing of semantic information whereas the posterior-dorsal LIFG (BA 44) seems to be specifically recruited for the use of phonological information [6,11,12]. On the other hand, there is evidence that the same LIFG regions are involved in the processing of phonemic and semantic verbal fluency tasks [13].

### Aims of the study

Meta-analytic methods allow the investigation of shared brain activation across individual studies by quantitatively identifying brain locations that are consistently associated with tasks or cognitive functions of interest. We applied the activation likelihood estimation technique as implemented in the GingerALE software [14-16] in order to study the brain activation during the processing of verbal fluency tasks.

As previous studies revealed contradictory results on whether the same or different brain regions are involved in the processing of semantic and phonemic verbal fluency tasks, we performed a coordinate-based meta-analysis separated for phonemic and semantic verbal fluency as well as a subtraction analysis of the activated brain regions in phonemic and semantic verbal fluency tasks. Based on previous results, we mainly expected cerebral activation during the processing of verbal fluency tasks in the left prefrontal lobe, particularly in the LIFG [7]. In a second step, we tested the hypothesized functional dissociation of the LIFG along semantic-phonological domain lines and expected that the posterior-dorsal LIFG is primarily involved in the processing of the phonemic verbal fluency tasks and the anterior-ventral LIFG in the processing of the semantic fluency tasks [4,6,11,12].

An important and very influential first systematic review of fMRI studies on verbal fluency [4] compared the activation patterns of semantic and phonemic verbal fluency tasks within the LIFG. The authors used bootstrap methods to calculate and compare the confidence intervals of the mean x-, y-, and z-coordinates between the two fluency tasks. The results support distinct dorsal-ventral locations for phonemic and semantic processes within the LIFG. Some individual studies comprised in this review included bilingual participants, right and left handed subjects as well as of individuals with right- and left-hemisphere dominance. Some previous fMRI studies suggested that bilingual individuals might have a greater increase in the blood oxygenation level-dependent signal in the LIFG (Brodman Area (BA) 45) than monolinguals [17]. Furthermore, there is evidence that left-handed individuals demonstrate a reverse speech organization in comparison to right-handed persons [18,19]. Right-hemisphere dominant language individuals on the other hand may exhibit a mirror reverse pattern of activation as compared to left-hemisphere dominant subjects [20]. In order to reduce variability and avoid issues of mixed language dominance, we restricted our analysis to right handed monolingual subjects. By reducing the inter-individual variation of the participants due to lateralization, handedness, and language background, and the inclusion of new original studies the current meta-analysis set out to replicate, validate and extend the results of the study of Costafreda and colleagues [4]. Here we applied the activation likelihood estimation (ALE) technique, which is a widely used, validated, automated and quantitative method for a voxel-wise meta-analysis of neuroimaging foci. Furthermore, we used subtraction analysis in order to directly compare the activation maps of phonemic and semantic verbal fluency tasks.

## Methods

### Included studies and participants

We performed a coordinate-based quantitative meta-analysis using the activation likelihood estimation (ALE) method (14-16, available at http://brainmap.org/ale/index.html). Results from neuroimaging studies of experiments on phonemic and/or semantic verbal fluency were included. The following inclusion criteria were used to select the studies: i) studies in peer-reviewed journals published in English; ii) use of active task-based functional MRI or PET neuroimaging techniques; iii) the sample consisted of right handed, healthy adult subjects of both sexes with a mean age ≤ 60 years; iv) results were reported using stereotactic three-dimensional coordinates; v) the field of view covered the whole brain.

### Search strategy for identification of studies

Peer-reviewed papers published in English were identified through PubMed, Cochrane Library, Embase, PsycLit, Biological Abstracts Dissertation Abstracts Online and Mental Health Abstracts using the search terms: “verbal fluency, phonemic verbal fluency, semantic verbal fluency, executive functions, and healthy control subjects. These terms were each combined (“AND”) with “functional magnetic resonance imaging” or “position emission tomography” or “fMRI” or “PET” or “neuroimaging” in order to identify the relevant functional imaging studies. We subsequently checked the reference sections of the publications that we found through our search, in order to identify additional studies that may have been missed. The search was conducted without any restriction of publication date or language used in the experiment. Direct e-mail communication with some researchers also provided additional data sets.

### Quality assessment

Two reviewers (SW, AT) independently conducted the literature search, assessed the methodological quality of the included trials and screened the studies for the above mentioned inclusion criteria. In case of disagreement between the reviewers, the disagreement was resolved by consensus discussion with one senior author of the research team (OT). To achieve a high standard of reporting, we adhered to the Preferred Reporting Items for Systematic Reviews and Meta-Analyses (PRISMA) guidelines and the revised Quality Of Reporting Of Meta-analyses (QUOROM) statement [21].

### Outcome measures

All studies used a block design including alternating blocks of the verbal fluency tasks with a baseline task (phonemic > baseline; semantic > baseline; for a detailed description of the baseline tasks see Tables 1 and 2). The tasks were presented auditory or visually during the investigation. Subjects were required to generate their responses overtly or covertly (see Tables 1 and 2). In all studies, subjects were asked to generate a word after they had heard an acoustic cue or seen a fixation cross on a monitor. In the phonemic verbal fluency task, subjects had to produce as many words as possible beginning with a specific letter. In the semantic verbal fluency task, a semantic category (e.g., animals or fruits) was presented instead of a letter. The participants were asked to generate an object name from the given category after each acoustic or visual cue.

**Table 1:** Studies included in the meta-analysis

	**First Author**	**Year**	**Sex (m/f)**	**Task**	**Paradigm**	**Template**	**Speech**	**Presen-tation**	**System**	**Threshold**	**Analysis**	**N (foci)**
1	Abrahams [22]	2003	14/4	p	Overt	Talairach	English	Auditory	1.5 T	p < 0.005 (uc)	XBAM	18 (22)
2	Bonelli [23]	2011	11/11	p	Covert	MNI	English	Visually	3 T	p < 0.05^3)^	SPM5	22 (4)
3	Brammer [24]	1997		p	Covert	Talairach	English	Auditory	1.5 T	p < 0.0001 (c)	XBAM	6 (6)
4	Curtis [25]	1998	5/0	p	Covert	Talairach	English	Auditory	1.5 T	p < 0.001 (c)	XBAM	5 (9)
5	Dye [26]	1999	6/4	p	Overt	Talairach	English	Auditory	PET	p < 0.05 (c)	SPM95	10 (10)
6	Fu^1)^[27]	2002	11/0	p	Overt	Talairach	English	Visually	1.5 T	p < 0.001 (uc)	XBAM	11 (29)
7	Halari^2)^[5]	2006	9/10	p	Overt	MNI	English	Auditory	1.5 T	p < 0.05 (c)	SPM99	19 (15)^7)^
8	Hutchinson [28]	1999	6/6	p	Overt	Talairach	English	Auditory	1.5 T	p < 0.05 (c)	SPM96	12 (6)
9	Lurito [29]	2000	2/3	p	Covert	Talairach	English	Visually	1.5 T	corrected		5 (15)
10	Nosarti^1)^[30]	2009	10/13	p	Overt	Talairach	English	Auditory	1.5 T	p < 0.001 (uc)	XBAM	28 (4)
11	Okada [31]	2003	8/2	p	Covert	Talairach	Japanese	Visually	1.5 T	p < 0.05 (c)	SPM99	10 (8)
12	Phelps [32]	1997	7/4	p	Overt	Talairach	English	Auditory	2.1 T	p < 0.005^4)^		11 (8)
13	Schlösser^2)^[33]	1998	6/6	p	Overt	Talairach	English	Auditory	1.5 T	p < 0.05 (c)	SPM99	12 (41)^8)^
14	Weiss [34]	2003	10/10	p	Covert	Talairach	German	Auditory	1.5 T	p < 0.001 (uc)	SPM99	20 (8)
15	Weiss [35]	2004	9/0	p	Covert	Talairach	German	Visually	1.5 T	p < 0.001 (uc)	SPM99	9 (8)
16	Audenaert [36]	2000	8/12	p/s	Overt	Talairach	Dutsh	Auditory	PET	p < 0.01 (uc)	SPM96	20 (13)
17	Heim [37]	2008	14/14	p/s	Overt	MNI	German	Visually	3 T	p < 0.05^3)^	SPM5	28 (4)
18	Kircher [38]	2011	15/0	p/s	Overt	Talairach	German	Visually	3 T	p < 0.001^5)^	SPM5	15 (27)
19	Meinzer [39]	2009	8/8	p/s	Overt	MNI	German	Visually	1.5 T	p < 0.05^6)^	SPM5	16 (14)
20	Meinzer [40]	2012	7/7	p/s	Overt	Talairach	English	Visually	3 T	p < 0.005^6)^	SPM5	14 (28)
21	Whitney [41]	2008	18/0	p/s	Overt	Talairach	German	visually	1.5 T	p < 0.05^3)^	SPM 2	18 (30)
22	Amunts [42]	2004	5/5	s	Covert	Talairach	German	Auditory	1.5 T	p < 0.05 (c)	SPM99	11 (8)
23	Basho [43]	2007	4/8	s	Covert	Talairach	English	Auditory	3 T	p < 0.05^5)^	AFNI	12 (6)
24	Gaillard [44]	2003	15/14	s	Covert	Talairach	English	Auditory	1.5 T	p < 0.0001 (c)	SPM99	29 (14)
25	Gurd [45]	2002	6/5	s	Covert	Talairach	German	Auditory	1.5 T	p < 0.001 (uc)	SPM99	11 (11)
26	Hwang [46]	2009	4/9	s	Overt/covert	Talairach	English	Auditory	3 T	p < 0.05^5)^	AFNI	13 (6)
27	Krug [47]	2011	64/32	s	Overt	Talairach	German	Visually	3 T	p < 0.05 (c)	SPM5	91 (5)
28	Ragland [48]	2008	7/6	s	Covert	Talairach	English	Visually	3 T	p < 0.05^5)^	SPM2	14 (7)

p: phonemic; s: semantic; uc = uncorrected; c = corrected;^1)^these studies seperately investigated the activation for easy and hard verbal fluency tasks, coordinates were included for easy tasks only;^2)^these studies seperately investigated the activation of females and males, coordinates were included seperately for males and females;^3)^family wise error rate corrected;^4)^ROI;^5)^Monte Carlo corrected;^6)^false discovery rate corrected;^7)^3 foci in females,^8)^12 foci in males;^4)^23 foci in females, 18 foci in males; MNI: Montreal Neurological Institut.

**Table 2:** Stimulus material of the included studies

	**First Author**	**Year**	**Task**	**Stimulus matrial phonemic**	**Stimulus matrial phonemic**	**Baseline condition**
1	Abrahams [22]	2003	p	t, a, b, g, f		Repetion of the word “rest”
2	Bonelli [23]	2011	p	a, s, w, d, e		Fixation of a cross
3	Brammer [24]	1997	p			Repetion of the word “rest”
4	Curtis [25]	1998	p	f, a, s		Repetion of the word “rest”
5	Dye [26]	1999	p	f		Repetion of nouns/verbs
6	Fu^1)^[27]	2002	p	t, l, b, r, s, c, p		Repetion of the word “rest”
7	Halari^2)^[5]	2006	p	f, a, s, p, r, w		Repetion of the word “rest”
8	Hutchinson [28]	1999	p	f, a, s, t, n		Count forward silently
9	Lurito [29]	2000	p	c		Fixation of a symbol
10	Nosarti^1)^[30]	2009	p	s, c, p, t, h, b, a		Repetion of the word “rest”
11	Okada [31]	2003	p	sa, ta, te		Repetion of the word “yasumi” (rest)
12	Phelps [32]	1997	p	English alphabet		Repetion of cue words
13	Schlösser^2)^[33]	1998	p	f, a, s, t, n		Count forward silently
14	Weiss [34]	2003	p	f, a, s, b		Silent rest
15	Weiss [35]	2004	p	f, a, s		Silent rest
16	Audenaert [36]	2000	p/s	n, a, k, b	Animals, jobs, fruit, vegetables, interior and furniture	Saying aloud month/days
17	Heim [37]	2008	p/s	f, b, k, m, sh, t	Birds, mammals, food, weapons, tools, toys	Production of any nouns
18	Kircher [38]	2011	p/s	f	Animals	Silent rest
19	Meinzer [39]	2009	p/s	f, a, h, n	Sports, fruits, body parts, musical instruments	Repetion of the word “Pause” (rest)
20	Meinzer [40]	2012	p/s	m, j, s, k, t, q, p	Body parts, types of music, clothing, insects, colors, spices, beverages, criminal acts	Repetion of the word “rest”
21	Whitney [41]	2008	p/s	s, w, d, b, h, e, a, f	40 German category	Repetion of the word “Pause” (rest)
22	Amunts [42]	2004	s		Flowers, furniture	Covertly produce month/days
23	Basho [43]	2007	s		Animals, academic subjects, body parts, car parts, colors, drinks, food, furniture, hobbies, musical instruments, occupations, shapes, sports, tools/appliances, things you wear, transportation	Repetion of the word “nothing”
24	Gaillard [44]	2003	s		Animals, food, cloths, furniture, toys, TV shows	Silent rest
25	Gurd [45]	2002	s		Fruits, cars, furniture	Covertly produce month/days
26	Hwang [46]	2009	s		Animals, colors, shapes, sports, tools, transportation, body parts, things you drink	Repetion of the word “nothing”
27	Krug [47]	2011	s		Vegetables	Reading aloud nouns
28	Ragland [48]	2008	s		Fruits, vegetables, furniture, vegetables	Repetion of the word “rest”

Outcome measures of the meta-analysis were the activation peaks of healthy control subjects during the processing of phonemic or semantic verbal fluency tasks. We included only those studies in our analysis, which reported activation as contrasted to a baseline condition in a healthy control group. Theoretical papers and reviews were excluded. Studies reporting combined group results and a region-of-interest analysis or only brain-behavior correlations, or did not report activation foci as 3-D coordinates in stereotactic space were excluded because these studies could not be meaningfully analyzed with ALE. Two studies reported results for men and women separately. In this case, we included the coordinates of males and females as two independent studies. In studies investigating easy and hard letters or categories, we selected the easy condition, because it was more comparable to the stimulus material in the other included studies.

### Statistical procedures

The X, Y, Z coordinates of every significant peak for all eligible contrasts constituted the input to the meta-analysis. All analyses were performed in the MNI reference space. Coordinates that were reported in Talairach space were converted to Montreal Neurological Institute (MNI) coordinates [49]. An ALE meta-analysis separated for phonemic and semantic verbal fluency was calculated according to the procedure described by Turkeltaub et al., [14] and Laird et al., [15], using the algorithm revised by Eickhoff et al., [16] and Turkeltaub et al., [50], which has been implemented in the GingerALE software. The ALE meta-analysis was executed using a random effects model (non-additive HBM, 22) implemented in GingerALE 2.1.1 (http://www.brainmap.org). A new algorithm was employed which consists of three steps resulting in an ALE map that is unbiased by the number of foci or contrasts included from each study [50,51]. The ALE algorithm delineates in which brain regions the convergence across all included imaging studies is higher than it would be expected if the results were independently distributed [16]. The three steps of the ALE analyses are as follows: (1) localization uncertainty is modeled for each focus of activity as a Gaussian distribution, the width of which is determined from the number of subjects of the study; (2) taking the union of the study-specific localization probabilities identified for each voxel yields the voxel-wise ALE value; (3) significance is tested using a random-effects method with a null hypothesis that the location of activation in each study is independent from the other studies [16]. Accordingly generated ALE maps were thresholded at p <0.05 using a False Discovery Rate (FDR) correction and a minimum clusters size of 100 mm^3^. For each of the resulting significant clusters, we additionally considered the number of studies that contributed to each. In order to ensure that the reported results represented coherence across multiple experiments, we eliminated ALE clusters and peaks that were based on less than three different studies [52,53]. Visualization of the results was implemented with MRIcron, using the Colin brain template in MNI space.

In a first step, we performed a ALE meta-analysis of all studies investigating phonemic (studies 1 to 21) verbal fluency tasks. After that we repeated this analysis with all studies using semantic verbal fluency tasks (studies 16-28). Results for phonemic verbal fluency tasks are shown in Table 3, for semantic fluency tasks in Table 4.

**Table 3:** Regions with significant activation during phonematic verbal fluency tasks

**Cluster**	**Anatomic label**	**Volume (mm**^**3**^**)**	**BA**	**x**	**y**	**z**	**ALE 10^**-3**^**	**N studies (foci)**
**1.**	Left frontal lobe. Inferior/middle frontal gyrus	16856	9	-50	12	24	35.23	23 (56)
			45	-48	28	14	25.32	
			44	-52	12	0	22.47	
	Left insula		9	-42	8	36	21.18	
			6	-54	2	46	15.99	
			13	-44	18	6	21.44	
**2.**	Left limbic lobe. Anterior cingulate gyrus	10744	32	-2	14	48	27.02	17 (34)
				-2	26	36	15.59	
	Right limbic lobe. Anterior cingulate gyrus		24	-6	28	22	15.29	
			32	4	32	34	17.63	
				6	22	28	12.54	
**3.**	Right insula	1304	13	38	26	-10	14.48	5 (6)
				44	16	-12	13.98	
	Right frontal lobe. Inferior frontal gyrus		47	52	18	-6	10.63	
			44	50	20	2	8.92	
**4.**	Left thalamus	960		0	-22	14	15.51	5 (5)
				-2	-18	6	12.29	
**5.**	Right cerebellum. Anterior lobe	576		36	-60	-32	13.67	4 (4)
**6.**	Left parietal lobe. Precuneus	552	7	-24	-54	62	15.36	3 (3)
**7.**	Right claustrum	520		30	22	-2	13.20	3 (4)
	Right caudate head			22	24	-4	9.71	
**8.**	Left putamen	384		-16	6	6	12.00	3 (3)

N: number of studies reporting at least one activation peak; Coodinates: MNI Space; mm^3^: cubic millimeter; BA: Brodman Area.

**Table 4:** Regions with significant activation during semantic verbal fluency tasks

**Cluster**	**Anatomic label**	**Volume (mm**^**3**^**)**	**BA**	**x**	**y**	**z**	**ALE 10^**-3**^**	**N studies (foci)**
**1.**	Left limbic lobe. Anterior cingulate gyrus	3312	32	-4	24	38	29.04	10 (12)
			8	0	26	52	11.50	
	Left frontal lobe. Superior frontal gyrus		6	-2	14	58	8.93	
	Left frontal lobe. Medial frontal gyrus		6	-6	12	54	8.86	
**2.**	Left frontal lobe. Inferior frontal gyrus	2256	45	-52	22	20	17.42	6 (8)
			9	-54	16	32	14.61	
**3.**	Left frontal lobe. Sub-gyral	1336	6	-24	6	52	17.69	5 (5)
**4.**	Left frontal lobe. Inferior frontal gyrus	1192	47	-32	24	-10	14.72	5 (5)
	Left claustrum			-34	20	-4	12.57	
**5.**	Left frontal lobe. Inferior frontal gyrus	840	47	-50	16	-2	14.58	4 (4)
				-50	24	-12	10.13	
				-46	24	-6	9.79	
**6.**	Left thalamus	784		-16	-2	12	17.71	3 (3)
**7.**	Left parietal lobe. Precuneus	728	7	-30	-68	48	16.48	3 (3)

N: number of studies reporting at least one activation peak; Coodinates: MNI Space; mm^3^: cubic millimeter; BA: Brodman Area.

**Figure 1:**
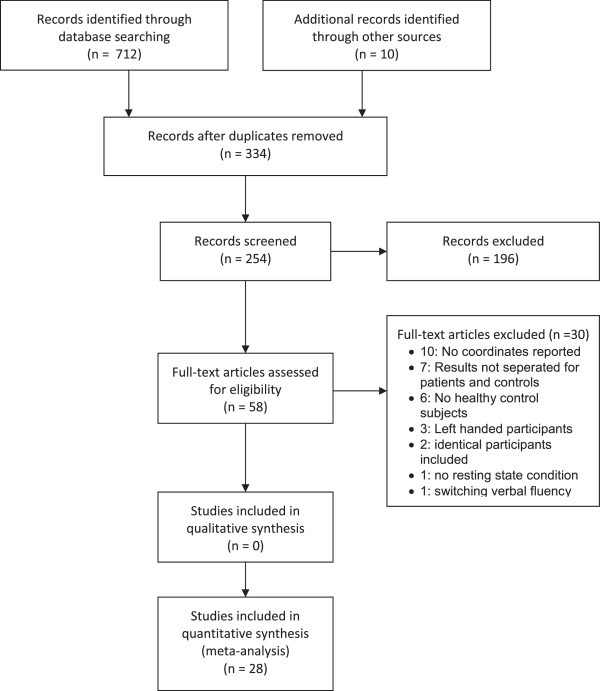
PRISMA flow chart of the literature search.

In a second step we determined the difference between the phonemic and semantic ALE images (Figure 1) by a subtraction analysis. The subtraction analysis allows a formal comparison of the difference between the two ALE maps (phonemic vs. semantic). For this purpose we used the thresholded NIfTI images from dataset A (i.e., “phonemic”, at p < 0.05), dataset B (i.e., “semantic”, at p < 0.05), and a pooled dataset A + B (i.e., “phonemic and semantic”, at p < 0.05). Regional differences between the phonemic and semantic verbal fluency tasks were tested by performing the ALE meta-analysis separately for each fluency task and computing the voxel-wise difference between the resultant ALE maps. Subtracting the ALE values calculated from semantic verbal fluency tasks from those calculated from phonemic fluency tasks gives a measure of the difference in convergence in the two maps.

Caution should be exercised when carrying out formal comparisons of ALE meta-analyses when the groups are disparate in total number of foci. In these cases, it is impossible to say with any certainty whether the difference maps reflect activation difference across groups of studies or simply show the effect of one group having a greater number of coordinates [54]. Because the number of studies and foci in the phonemic verbal fluency task was significantly higher than in the semantic verbal fluency task, the resulting difference in power between the two tasks may affect the results of the subtraction analysis. In order to improve the sensitivity of the subtraction analysis and increase the number of studies and foci included in the semantic verbal fluency map, we added the coordinates of the semantic verbal fluency tasks included in the six studies which investigated both phonemic and semantic verbal fluency to the semantic part of the subtraction analysis. Thus, data set A (“phonemic”) of the subtraction analysis included studies 1 to 15, data set B (“semantic”) studies 16 to 28 and the pooled data set studies 1 to 28.

A prerequisite of the subtraction analysis of GingerALE is that the number of included studies, subjects and foci of the pooled data set (A + B) has to be the exact sum of the number of studies, subjects and foci of data sets A and B. If we had included the coordinates of the phonemic verbal fluency tasks of the six studies which investigated both phonemic and semantic verbal fluency in the subtraction analysis as well, this prerequisite would have no longer been fulfilled and it had not been possible to execute the subtraction analysis.

A minimum of at least 8 to 10 experiments should be included in an ALE meta-analysis in order to get valid results (Prof. S. Eickhoff, author of GingerALE, personal communication). According to the failure report of GingerALE, the subtraction analysis should include a minimum of at least 15 studies in each data set in order to provide enough statistical power. Thus, the small number of included studies, specifically in the analysis of the semantic verbal fluency map, must be kept in mind when interpreting the results of our subtraction analysis.

## Results

The search identified 254 studies which were screened by title and abstract. 196 of these studies had to be excluded because they did not fulfill the inclusion criteria. The full text of the remaining 58 studies was scrutinized by two independent reviewers (SW, AT). Thirty of these studies were excluded from analyses: ten studies because the y-, x-, and z-coordinates were not reported, seven because the results were not separated for patients and controls, six studies did not include healthy control subjects, two included identical participants as Krug et al. 2011, one study had no baseline condition and one included a switching verbal fluency task. Finally, 28 studies investigating healthy volunteers were included in our meta-analysis. Fifteen studies assessed phonemic verbal fluency, seven studies semantic verbal fluency, and six studies both phonemic and semantic verbal fluency (Figure 1).

Across the included studies, 490 healthy control subjects (60% men, 40% women) were analyzed. Mean age of the included participants was 30.8 years (range: 21 – 56.7 years). The 21 studies assessing phonemic verbal fluency yielded 245 foci inside the MNI space; the 13 studies investigating semantic verbal fluency performance yielded 117 foci. The coordinates of these studies were entered into two separate ALE meta-analyses to test for regional concordance within phonemic or semantic verbal fluency as well as in a subtraction analysis in order to assess significant differences in the brain activation during phonemic and semantic verbal fluency tasks.

### Phonemic verbal fluency

The search identified 21 studies comprising 23 experiments investigating the brain activation during phonemic verbal fluency tasks, including 307 healthy volunteers. The meta-analysis of the coordinates revealed one cluster covering the LIFG/LMFG (BA 6, 9, 44 & 45) and left insula (BA 13) as well as an additional cluster located in the left and right anterior cingulate gyrus (BA 24, 32) as well as one in the right insula and frontal lobe (BA 44, 47). A further cluster revealed activation of the left thalamus. Additionally, we found several brain regions of concordance located in the left precuneus (BA 7) and putamen as well as in the right Claustrum and Caudate Head.

### Semantic verbal fluency

**Figure 2:**
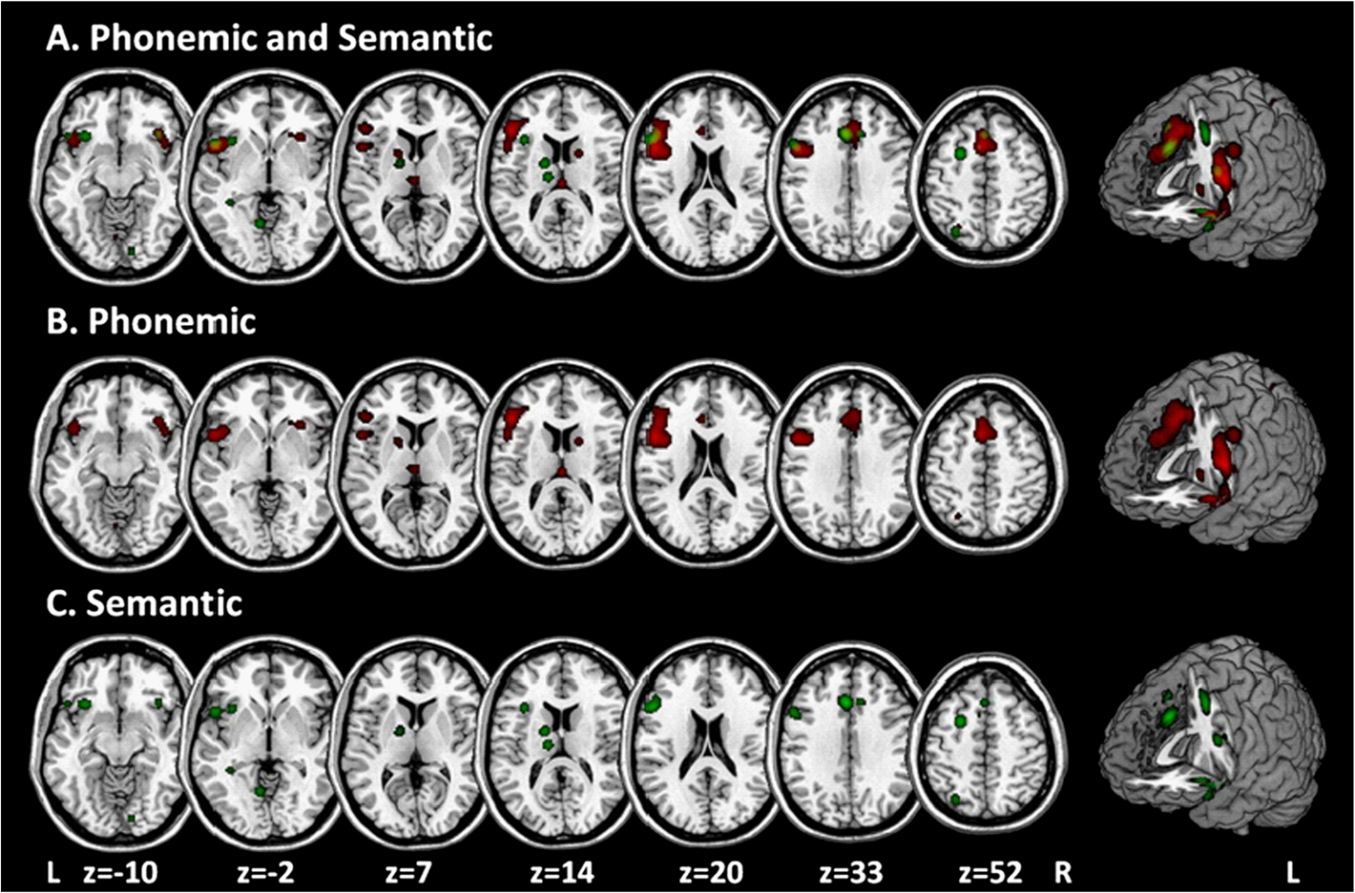
**Shows the significant cluster of brain activation in the processing of verbal fluency, threshold: p <0.05 using a False Discovery Rate (FDR) correction and a minimum clusters size of 100 mm3.** Data are shown in neurological convention (R = R, L = L; R: right; L: left).

Thirteen studies including 292 healthy volunteers were analyzed. The meta-analysis of the coordinates revealed clusters of brain activation in the left anterior cingulate gyrus (BA 32) as well as in the left superior (BA 6, 8) and medial frontal gyrus (BA 6) (Figure 2; Table 4). Furthermore, we found brain activation in the LIFG (BA 9, 45) as well as one cluster covering the left claustrum and LIFG (BA 47). Additional clusters comprised the LIFG (BA 47), the left Thalamus and Precuneus (BA 7).

### Subtraction analysis

A subtraction analysis of the experiments using phonemic and semantic verbal fluency tasks was performed. The phonemic fluency data set included 17 experiments reporting 193 foci (N = 196), the semantic data set 13 experiments reporting 117 foci (N = 298) and the pooled data set 30 experiments yielding 310 foci (N = 494). The subtraction of the phonemic versus semantic verbal fluency map revealed no significant differences in the ALE maps.

## Discussion

In this study, we report the results of a coordinate-based ALE meta-analysis of the brain activation during phonemic and semantic verbal fluency tasks in healthy volunteers. The main clusters of brain activation were seen in the left frontal lobe, specifically the IFG, MFG and medial frontal gyrus (BA 6, 9, 44, 45, 47), as well as in the anterior cingulate gyrus (ACC) (BA 24, 33). These results confirm previous studies suggesting that brain regions primarily in the left prefrontal gyrus, particularly in the LIFG and LMFG, are involved in word production and speech processing in verbal fluency tasks [4,55-60]. Regarding the ACC, phonemic verbal fluency tasks predominantly activated the left (BA 32, 24) and right ACC (BA 32), semantic verbal fluency tasks only the left ACC (BA 32). This is in line with previous studies suggesting that the cingulate gyrus (BA 32, 24) is activated during word generation and may therefore reflect the attentional demands of verbal fluency tasks [43,55,60]. Patients with bilateral anterior cerebral infarction for example often suffer from akinetic mutism and verbal fluency deficits. Furthermore, blood flow in the anterior cingulate gyrus (BA 24) increases during the processing of single words or letters [61].

The left parietal precuneus (BA 7) was activated in the processing of phonemic and semantic fluency tasks. The precuneus (BA 7) is involved in phonemic discrimination and working memory [56,57,62,63] and was repeatedly associated with the processing of phonological information. Furthermore, this region plays a central role in visual attention of stimuli and speech.

Further cluster of activation included the left and right insula, left Thalamus and Putamen as well as the right Claustrum and Caudate Head. Another cluster of activation was seen in the cerebellum. There is evidence that the (left) sub-lobar insula is involved in speech processing and the execution of verbal fluency tasks [64]. Specifically the left anterior insula has been suggested to be involved in the articulatory planning of orofacial movements [65]. A systematic review of Price reports that speech production leads to an increased activation in the cerebellum, the anterior insula as well as in the left Putamen [66]. The ACC and head of caudate have been found to be involved in word selection. The initiation and execution of movements during speech production increase the activation in the left putamen. The thalamus has also been shown to be involved in the processing of verbal fluency [67].

### Brain activation in the processing of phonemic versus semantic verbal fluency tasks

As can be seen in Tables 3 and 4, Brodman area 44 was only involved in the processing of phonemic verbal fluency tasks, whereas BA 9, 45 and 47 were activated in phonemic and semantic verbal fluency tasks. The result that BA 44 was only involved in the processing of phonemic verbal fluency tasks is in line with previous studies which suggested that the posterior-dorsal LIFG (BA 44) is specifically involved in the processing of phonemic information [6,11,12]. Phonemic fluency is most likely triggered by subvocal syllabification that overlaps with processes of inner speech such as motor programming and articulation, as indicated by stronger activations of posterior LIFG (BA 44; Figure 2, blue) close to adjacent (pre)motor areas [68].

Contrary to the hypothesis that the anterior-ventral LIFG (BA 45, 47) is specifically involved in the processing of semantic information, BA 45 and 47 were activated in the processing of semantic and phonemic verbal fluency tasks. Previous studies also revealed an activation of BA 45 and 47 in the processing of phonemic and semantic verbal fluency tasks [3,37,42]. These results are consistent with assumptions that phonological search processes are not exclusively based on phonemic information, but may also rely on semantic facilitation [68]. A variety of previous studies failed to find evidence for the hypothesis that semantic processing preferentially activates anterior ventro-lateral regions of the PFC when compared to phonological processing [13,69-72]. A recent study directly comparing phonemic vs. semantic verbal fluency tasks while controlling for the effects of task demand implies that activity in the anterior-ventral LIFG (BA 45) is mainly related to task demand and individual ability [68]. In summary, our results support the hypothesis that the posterior LIFG is specialized for the use of phonemic material but failed to confirm the hypothesis that the anterior LIFG is specifically involved in the processing of semantic information.

The subtraction analysis revealed no cluster of significantly greater activation during the processing of phonemic than during semantic verbal fluency tasks. Previous studies suggested that caution should be exercised carrying out formal comparisons of ALE meta-analyses when the two data sets are disparate in the total number of foci. In these cases, it is impossible to say with any certainty whether the difference maps reflect activation difference across groups of studies or simply show the effect of one group having a greater number of coordinates [54]. In order to improve the sensitivity of the subtraction analysis and increase the number of foci included in the semantic verbal fluency map, we added the coordinates of the semantic verbal fluency tasks included in the six studies which investigated both phonemic and semantic verbal fluency to the semantic part of the subtraction analysis. Thus, data set A (“phonemic”) of the subtraction analysis included 15 studies, data set B (“semantic”) 13 studies. In our previous analysis, the subtraction analysis revealed a greater activation during phonemic than semantic verbal fluency tasks in a cluster in the left LIFG. Due to the inclusion of two additional semantic verbal fluency studies in the analyses of the revised manuscript, the subtraction analysis did not longer reveal different activation patterns in phonemic compared to semantic verbal fluency tasks. The observed difference of activation in the previous analysis may be the result of power differences between the two tasks, because the number of the included studies and foci in the phonemic verbal fluency task was significantly higher than in the semantic verbal fluency task. The fact that the clusters of activation in the left hemisphere coincided in phonemic (Table 3) and semantic (Table 4) verbal fluency tasks except for BA 24 (ACC) and 7 (Putamen) substantiate this assumption.

Previously, a domain specific activation in the left posterior temporal cortex near the middle temporal gyrus for semantic processing was found [9,11,12,60]. In the current meta-analysis, activation of the left temporal gyrus in the processing of semantic verbal fluency tasks as previously reported [10-12,43] could not be replicated. This may be due to the lower overall activation in semantic verbal fluency tasks and the lower number of semantic verbal fluency studies included in the meta-analysis.

### Limitations

The studies included in our meta-analysis differed regarding design, methodology, and the study population. As shown in Tables 1 and 2, the included studies differ in their stimulus material, baseline condition and language (English, German, Dutch or Japanese) as well as in the kind of stimulus presentation (auditory versus visually) and response generation (overt versus covert). These differences might have affected the results of our analysis.

The included experiments used two different types of baseline conditions. Twenty-two of the 28 experiments (15 of 21 phonemic and 2 of 13 semantic fluency experiments) involved a covert or overt repetition of a given word (“rest”) or of a familiar sequence (e.g., forward counting, days of the week or month of the year). The performance of such standardized language production requires at least some low-level phonologic processing. When subtracted from the experimental tasks, they would at its best attenuate phonologic activity in the final images, which is most likely localized to the more posterior and dorsal areas of the LIFG [6,73]. Consequently, most phonemic experiments may have underestimated the extent of phonologic activity. The second type of baseline condition was a passive task, such as silent rest or visual fixation of a cross or symbol. There is some evidence that a functionally connected brain network including the LIFG might be associated with resting states [74-76]. In this framework, the effects would be opposite to those using a standardized language production baseline task. Semantic activity would then be underestimated in semantic experiments, which used a resting state (two of thirteen semantic experiments) baseline condition.

The performance in verbal fluency tasks depends on the difficulty of the stimulus material. As can be seen in Table 2, the majority of English and German phonemic verbal fluency studies used variations of the COWAT stimulus material (FAS) additional to further letters. The use of different stimulus material could have affected our results because previous studies suggested that the CFL subtest of the COWAT is more difficult than the FAS subtest [77]. Lacy and colleagues [78] on the other hand revealed a comparable performance in the two forms of the COWAT. Borowski and colleagues [79] investigated the association between different letters and their difficulty. Based on the frequency of the generated words, the authors categorized H, D, M, W, A, B, F, P, T, C, S as easy English letters, I, O, N, E, G, L, R as moderately difficult letters and Q, J, V, Y, K, and U as hard letters. According to this classification, the included studies of our meta-analysis only used easy to moderate letters. The most frequently used categories were furniture (6 of 13), animals (5 of 13) as well as fruits, food, body parts (4 of 13) and vegetables, cloths and colors (3 of 13).

The majority of the participants in the current meta-analysis were native English or German speakers, one study consisted of Dutch participants, one of Japanese individuals. The fact that different languages use different strategies for encoding grammatically information leads to the question whether an unitary network of brain regions specialized for processing grammar in a broad sense is involved in the processing of different human languages, or whether different languages impose distinct processing demands relying on non-identical neural mechanisms. Previous studies on language dependent processing of verbal material suggest that different brain networks are involved in the processing of different languages [80,81]. The language of the included studies might accordingly have affected the activation patterns. However, a secondary analysis excluding the Dutch and Japanese studies revealed the same results than the first analysis. Furthermore, Oberg and Ramirez [82] suggested that as long as the letter frequency was considered, the number of generated words were remarkably similar across different languages.

Subjects generated covert responses in 7 of 21 phonemic studies and 6 of 13 semantic, verbal fluency tasks. Whereas overt paradigms hold a risk to produce movement artifacts, covert verbal responses do not allow to determine whether the subjects perform the task as instructed and to assess the task performance [55]. Because of the differences between covert and overt verbal fluency paradigms, it seems to be difficult to generalize the results from covert response paradigms to overt response paradigms. Furthermore, it is possible that the cognitive processes operating during covert verbal responding are different in some aspects to those operating during overt verbal responding. Although a direct comparison of overt and covert responses in a stem completion task showed greater LIFG activation with overt than covert responses, the location of the peak of activation did not differ [83]. In order to clarify the effect of the response generation on the brain activation, we statistically compared the two sets of foci by subtracting the ALE maps of the overt and covert verbal fluency tasks. The covert fluency data set included 14 experiments reporting 122 foci, the overt data set 17 experiments reporting 255 foci and the pooled data set 31 experiments yielding 377 foci. The subtraction of the covert versus overt verbal fluency maps resulted in a higher activation likelihood in the LIFG (BA 46; X: -52; Y: 27; Z: 17; 1816 mm^3^). No significant differences were seen subtracting the overt from covert verbal fluency map. The auditory presented fluency data set included 18 experiments reporting 184 foci, the visually presented data set 13 experiments reporting 178 foci and the pooled data set 31 experiments yielding 362 foci. Auditory presentation of the stimuli resulted in a significantly greater activation in the left medial frontal gyrus (BA 8; 312 mm^3^) and left Insula (BA 13, 160 mm^3^).

Regarding gender differences in the performance of verbal fluency tasks, previous studies revealed heterogeneous results. Among the functional imaging verbal fluency studies focusing on sex differences, no study has highlighted a statistically significant behavioral difference between groups of men and women. No activation difference was found for men and women selected either on the basis of a same high level of VF performance or on differential cognitive performances (5; 60). Furthermore, a variety of non-imaging studies investigating the verbal fluency performance of healthy subjects also did not found differences between men and women in their phonemic or semantic verbal fluency performance [84-87]. On the other hand, Gauthier and colleagues [88] reported sex effects in a sample of high performers in seven cortical structures (the left ITG, anterior and posterior cingulate, right ACC, SFG, dlPFC and lingual gyrus) during the processing of a phonemic verbal fluency paradigm. The majority of the included studies in our meta-analysis investigated the brain activation during verbal fluency tasks in a sample of men and women without consideration of gender (Tables 1 and 2). Thus, we were not able to identify the activation patterns separately for men and women.

In conclusion, the aim of our meta-analysis was to compare the brain activation during the processing of semantic and phonemic verbal fluency. Tables 1 and 2 show that the number of studies using German or English language, a visually or auditory presentation of the stimulus material or an overt or covert paradigm was comparable between the studies investigating phonemic or semantic verbal fluency. Thus, we would suggest that the effect of these confounding variables on brain activity was equally distributed in phonemic and semantic verbal fluency tasks. Nevertheless, future studies are needed which investigate the brain activation during verbal fluency tasks separately for studies using different designs with respect to stimulus presentation, language or response generation, respectively. The number of included studies in our meta-analysis, specifically in the analysis of semantic verbal fluency tasks, was too small to compare subgroups of studies presenting the stimulus material visually or auditory or using overt or covert paradigms.

Coordinate-based neuroimaging meta-analyses usually pool studies that have different statistic thresholds. As can be seen in Tables 1 and 2, the statistical threshold of the individual studies of our meta-analysis ranges from strict family-wise error rate correction to uncorrected p-values of p < 0.005. Assuming equality across studies irrespective of their statistical threshold could have the consequence of giving more weight to studies using less strict statistical thresholds, as these are likely to report more significant findings than studies using stricter statistical thresholds [86]. In our meta-analysis, six studies used an uncorrected statistical threshold. Additional file 1: Table S1 and S2 show the number of foci from each experiment contributing to the significant clusters of phonemic and semantic verbal fluency. With 9 and 5 foci, respectively, the study of Abrahams and colleagues [87] contributed by far the most foci to cluster 1 and 2 of phonemic verbal fluency. However, in the other five studies with uncorrected statistical thresholds, it could not be observed that they report more significant findings than the studies using stricter statistical thresholds.

A further limitation of our meta-analysis might be that the power of the analyses cannot be aggregated across the included studies, because the GingerALE software is not suited to correct for false negative results [89]. On the other hand, this also means that ALE minimizes the risk of false positive results and is not susceptible for outlier effects. Meta-analytic results are often influenced by the heterogeneity of the included studies. Therefore, it is an aim of meta-analysis to statistically control for potential sources of heterogeneity. The ALE software did not allow the investigation of heterogeneity between the individual studies; therefore, we cannot fully exclude that the results might be influenced by a possible heterogeneity of the individual studies. Nevertheless, we tried to minimize the heterogeneity through the definition of relatively strict inclusion criteria. Furthermore, the new ALE algorithm is based on a random effects model, which is more conservative than the fixed-effects model and incorporates both within-study and between study variance.

## Conclusion

The current meta-analysis investigated the brain activation of healthy volunteers during phonemic and semantic verbal fluency tasks. Our analyses corroborate the involvement of the left inferior/middle frontal gyrus (BA 6, 9, 45, 46, 48) as well as the ACC in the processing of verbal fluency tasks. Our comparison of brain activation during the execution of either phonemic or semantic verbal fluency tasks revealed evidence for spatially different activation patterns in the posterior but not the anterior regions of the LIFG/LMFG during phonemic and semantic verbal fluency processing.

## Competing interests

AT has received research funding from Rules based medicine Inc., Austin, Texas, USA. None of the other authors of this study has business or personal interests in industrial enterprises since 1. November 2009. OT and AS were supported by a Federal Ministry of Education and Research Grant 01GW0730 to KL and OT.

## Authors’ contributions

SW and AT conducted the literature search, assessed the methodological quality of the included trials and screened the studies for the above mentioned inclusion criteria. AS created the figures of the manuscript. OT was involved in the analysis and interpretation of the data and writing of the manuscript. All authors critically read and approved the final version of the manuscript. The corresponding author had final responsibility for the decision to submit for publication.

## Supplementary Material

Additional file 1 Coordinates of the included studies separated for phonemic and semantic verbal fluency tasks (values with two decimal places are due to conversion from Tailarach to MNI coordinates). Click here for file
