# Supplementary material for: A coordinate-based ALE functional MRI meta-analysis of brain activation during verbal fluency tasks in healthy control subjects
Source: BMC Neurosci. 2014 Jan 24;15:19. doi: 10.1186/1471-2202-15-19 (PMC3903437; doi:10.1186/1471-2202-15-19)
Supplement: Additional file 1 — Coordinates of the included studies separated for phonemic and semantic verbal fluency tasks (values with two decimal places are due to conversion from Tailarach to MNI coordinates). [file 1471-2202-15-19-S1.docx]

**Coordinates of the included studies separated for phonemic and semantic verbal fluency tasks (values with two decimal places are due to conversion from Tailarach to MNI coordinates)**

**Phonemic verbal fluency tasks**

Kircher, 2011 (N=15)

-24.94 -63.57 -21.4

29.13 -69.55 -19.49

-6.89 12.25 53.71

8.24 12.3 53.45

-41.85 6.99 22.33

-32.71 6.4 61.45

-54.51 1.58 45.49

60.99 7.84 37.3

64.98 4.3 10.69

69.19 4.55 1.63

67.01 -22.98 -5.69

73.57 -24.56 -0.05

Audenaert, 2000 (N=20)

-41.29 29.4 -13.52

-54.01 11.46 1.92

-4.97 -21.29 27.86

-5.19 13.19 17.73

20.97 21.53 36.63

-57.18 -63.5 -7.41

66.11 -52.24 2.82

Weiss, 2003 (N=20)

-42.11 14.9 3.62

-50.62 37.8 19.43

44.15 13.96 -11.2

52.83 18.66 -7.33

5.92 18.81 42.76

-6.97 14.92 47.84

31.03 -65.99 -38.93

3.11 -78.02 -28.3

Dye, 1999 (N=10)

-35.34 12.74 26.13

-39.8 35.71 19.45

-35.72 38.32 1.2

-37.53 21.25 25.33

5.92 18.81 42.76

3.65 33.27 36.88

-4.8 25.98 51.19

9.69 -90.34 -22.71

-13.93 -10.53 5.66

-48.71 -60.35 -20.2

Phelps, 1997 (N=11)

-19.01 46.58 42.68

-23.31 35.72 41.58

-48.51 28.68 13.57

-2.81 26.87 37.63

5.67 22.36 23.36

-11.62 22.3 23.66

-45.26 34.21 15.21

5.77 29.67 32.72

Schlösser, 2003: females

(N=6)

-55.1 34.41 6.39

-50.49 12.48 24.17

-50.6 18.24 16.88

-2.56 22.15 56.02

-52.25 -26.07 48.19

-43.61 -30.5 46.24

41.88 26.3 -16.87

-23.94 -52.79 59.32

27.59 -70.41 28.8

24.76 -66.68 -23.06

35.48 -60.68 -28.32

6.25 -50.97 54.14

-6.81 -40.8 48.87

-8.92 -31.68 54.73

-52.22 -63.06 42.88

-45.9 -48.98 32.42

-45.84 -57.28 35.48

60.1 -58.62 38.29

64.25 -51.13 26.27

51.29 -46.72 28.3

69.87 -5.05 -32.17

59.16 -15.3 -26.49

69.76 0.71 -39.46

Schlösser, 2003: males (N=6)

-50.52 18.87 23.54

-48.5 33.14 15.37

-4.82 25.77 48.97

-23.81 -62.81 67.03

-23.9 -54.71 61.75

-28.26 -44.09 60.77

41.79 -61.9 -41.75

52.69 -69.96 -36.66

37.39 -84.44 -52.9

72.57 -14.06 9.02

72.74 -12.82 22.34

70.7 -14.12 31.47

-4.62 -42.71 51.27

-2.46 -33.99 52.61

-0.4 -45.65 42.52

-56.74 -55.82 28.8

-58.74 -52.45 41.94

-71.88 -29.72 33.2

Abrahams, 2003 (N=18)

-48.3 3.98 24.98

-48.26 11.95 29.79

-32.31 39.58 14.46

5.85 12.76 35.51

-39.52 9.41 36.62

-2.7 5.8 41.95

13.3 18.55 28.09

-2.62 6.43 48.61

-44.23 30.51 9.96

-36.17 1.43 42.95

-36.04 -11.77 50.98

-13.85 -4.48 12.9

-50.8 12.33 -0.46

-40.01 34.15 2.8

-6.26 28.59 21.83

-44.43 19.17 -7.97

-40.19 21.86 -13.91

34.77 36.18 20.38

-50.73 12.85 5.09

-3.09 -73.73 -5.08

-13.96 2.34 5.51

-19.89 -77.81 32.59

Lurito, 2000 (N=5)

-61.32 -55.57 8.68

-59.22 -31.96 8.55

-48.71 -54.93 -19.61

-44.54 -58.91 52.43

-33.84 -58.54 44.36

-48.96 -45.41 47.81

-3.76 15.79 45.46

-3.53 -1.05 60.57

-56.19 16.77 1.44

-28.23 -61.76 -24.89

51.16 -46.59 18.2

44.32 25.09 4.5

39.29 39.88 36.74

36.54 -64.07 -30.24

30.79 -67.86 -58.91

Brammer, 2000 (N=6)

1.87 -59.06 48.29

-45.09 12.5 24.08

-45.03 16.21 29.31

-41.69 11.53 36.44

-26.19 -65.54 49.41

1.56 20.71 40.4

Hutchinson, 1999 (N=12)

-0.54 27.71 46.47

-39.68 10.39 24.2

-48.43 24.84 18.43

1.45 35.18 34.49

-26.06 -54.72 61.78

35.42 -58.97 -32.97

Fu, 2001 (N=11)

-12.04 59.68 -1.32

-1.69 -72.41 20.54

-39.97 49.77 9.1

-34.34 48.26 27.08

46.56 37.73 13.3

50 31.54 27.3

-53.79 6.53 18.1

46.5 38.38 8.75

27.98 22.93 -6.21

38.88 37.94 4.44

-0.87 54.76 25.87

7.86 43.4 30.21

-64.36 -55.19 24.38

-61.38 -58.22 3.34

-63.33 -56.67 20.03

50.87 -47.71 -5.21

-61.44 -60.87 0

-68.65 -49.48 28.37

-55.59 4.87 46.31

16.37 7.43 12.33

29.1 23.24 -2.9

2.91 -73.61 41.87

-26.02 -81.72 59.97

-30.12 -87.11 -8.9

24.89 -79.89 -15.03

22.8 -82.67 -10.24

2.51 -86.4 8.4

24.85 -83.49 -19.16

-43.24 -77.35 -18.61

Okada, 2003 (N=10)

-40.09 16.1 -6.62

-44.97 7.91 32.38

-39.73 4.56 19.17

-2.02 -20.16 5.29

-7.54 -29.83 -5.99

5.75 19.69 29.23

9.99 27.9 24.98

-5.17 29.76 22.81

Curtis, 1998 (N=5)

4.97 18.67 52.87

-48.18 13.63 36.35

-45.41 13.41 -0.66

-4.81 -48.52 35.03

-33.15 16.25 29.11

-39.86 36.36 14.91

47.48 20.12 -2.9

-41.85 12.51 24.02

-36.03 3.64 55.06

Nosarti, 2009 (N=26)

13.17 -69.97 -0.13

-46.31 26.77 15.97

1.68 13.89 47.8

20.45 25.34 -2.96

Heim, 2008 (N=28)

-52 6 23

-44 28 23

Halari, 2006 (N=9), males

-2 14 54

10 14 58

6 28 30

-50 0 48

-52 8 34

-52 14 20

6 -32 8

-4 -36 0

2 -16 6

-4 -72 -10

6 -70 -8

-18 8 1

Halari, 2006: women (N=10)

-42 18 -6

-12 4 20

44 18 -12

Weiss, 2004 (N=9)

-42 6 36

-22 12 54

52 20 4

46 10 32

44 44 27

-4 22 36

-26 -64 54

-8 -18 4

Bonelli, 2011 (N=22)

-44 22 24

-30 24 0

32 22 0

-28 20 -10

Meinzer, 2009 (N=16)

-50 14 -3

-53 10 30

-18 9 63

-15 -78 12

0 -23 15

-36 -41 2

33 -38 7

**Semantic verbal fluency tasks**

Ragland, 2008 (N=14)

-28.12 52.9 7.47

46.69 40.89 24.19

-1.41 13.96 60.17

40.4 10.55 32.9

-34.65 20.79 -2.69

36.58 16.06 -11.28

39.71 -52.57 -33.68

Gurd, 2002 (N=11)

-2.8 24.74 37.84

10.07 26.29 30.74

-33.3 44.66 22.94

37.94 53.21 18.64

-38.08 23.68 -17.49

41.85 28.22 -19.3

-37.48 8.48 26.59

9.43 -87.96 -43.11

Kircher, 2011 (N=15)

-24.94 -63.57 -21.4

-6.89 12.25 53.71

9.89 -86.66 -6.27

8.26 12.51 55.67

-68.99 -24.65 6.87

-50.18 -12.55 43.45

-66.93 -13.36 1.24

-26.04 -68.75 60.93

-28.36 -64.48 49.34

-17.55 -55.62 51.64

-17.38 -19.41 71.59

-41.42 -82.79 37.93

-42.51 -5.55 -31.33

-50.57 22.81 19.79

-54.77 20.43 29.06

Krug, 2011 (N=91)

-30.95 -56.76 17.25

-37.71 -40.26 -1.08

5.32 -5.43 -9.74

-29.35 23.27 -10.87

-7.12 28.02 38.71

// Basho, 2007 (N=12)

-12.73 -15.11 13.93

-16.04 -3.63 10.61

-35.53 21.17 12.97

-2.81 23.57 36.83

-24.18 8.89 53.22

-8.47 -60.47 -0.7

Gaillard, 2003 (N=29)

-5.06 -89.82 6.63

-54.71 15.43 32.92

-28.43 20.35 61.12

-50.99 24.05 -12.82

-0.47 26 51.12

-30.53 -68.95 47.58

-31.51 -45.61 -24.19

-25.99 -73.86 63.68

59.51 42.03 12.65

51.09 21.77 26.01

46.05 29.53 -28.46

-5.86 57.41 -25.86

34.15 28.66 54.75

-36.06 51.13 -22.48

Amunts, 2004 (N=11)

-56.72 -0.98 41.3

53.38 3.04 32.3

-35.72 16.43 -3.36

35.53 29.44 -5.86

-31.56 -61.33 -31.6

34.07 -55.08 31.66

37.96 42.37 17.47

-26.17 -70.86 49.93

Heim, 2008 (N=28)

-52 2 23

-44 26 25

Meinzer, 2009 (N=16)

-50 14 -3

-42 7 27

-21 -75 12

-6 17 43

-6 28 32

36 -35 -3

-18 9 60

Meinzer, 2012 (N=20)

-47.15 12.16 31.99

-1.79 31.88 33.75

-50.75 -46.35 -8.1

-27.36 -64.04 42.56

20.51 21.4 0.79

39.81 29.14 -9.27

-5.03 -90.68 8.95

-14.55 -91.39 24.87

Audenaert, 2000 (N=20)

-44.48 49.47 -5.36

-50.84 17.54 -2.09

-57.03 19.7 21.33

23.9 22.55 12.95

-4.93 -20.97 31.19

39.71 59.03 -11.1
